# Pickleworm (*Diaphania nitidalis* Cramer) Neonate Feeding Preferences and the Implications for a Push-Pull Management System

**DOI:** 10.3390/insects7030032

**Published:** 2016-07-05

**Authors:** Rosalie Leiner, Helen Spafford

**Affiliations:** Department of Plant and Environmental Protection Sciences, University of Hawaii, Manoa, 3050 Maile Way, Honolulu, HI 96822, USA; rcleiner@gmail.com

**Keywords:** push-pull cropping, trap cropping, Crambidae, Lepidoptera, Cucurbitaceae, host plant preference

## Abstract

Push-pull cropping approaches for pest management target the oviposition behavior of adult females. However, insect larvae may move from the natal host and undermine the effectiveness of this approach. We investigated the longevity and feeding preference of pickleworm neonates (*Diaphania nitidalis* Cramer (Lepidoptera: Crambidae)) in relation to a potential push-pull cropping approach incorporating squash as a trap crop (pull) and watermelon as a deterrent intercrop (push) to protect a main crop of cantaloupe. Neonates could survive between 24 to 64 h without food, indicating they have some initial energy reserves to keep alive while in search of a suitable feeding site. To assess neonate feeding preferences, naive neonates were given the choice of five foods; leaves of squash, cantaloupe, watermelon, bean, and a pinto bean-based artificial diet. To assess if previous feeding experience influences neonate food source preference, neonates were allowed to feed on one of the five foods for 24 h and then given the same choice of the five food sources. The neonates, with or without previous feeding experience, did not appear to have a significant preference for any of the cucurbits: squash, cantaloupe, or watermelon, but they did prefer a cucurbit to the bean leaf or artificial diet. Feeding experience on one of these non-host foods made neonates more accepting of these food sources in the choice arena even when host plant food sources became available. It appears that neonate feeding preferences of pickleworm would neither hinder nor enhance the potential success of the proposed cucurbits to be used in a potential push-pull cropping approach for pickleworm management.

## 1. Introduction

Worldwide there has been an increased interest in alternative pest management strategies to reduce pesticide use and create more environmentally sound production systems. One such alternative is an ecological management approach called a “push-pull strategy” [1,2]. One form of this approach employs the use of a trap crop (pull) and a deterrent intercrop (push) to effectively prevent a pest from settling on the main crop [1]. This strategy has been used in some systems to target Lepidoptera pests and reduce larval feeding damage on the main crop. An example of this cropping approach is the effective management of the stemborer, *Chilo partellus* Swinhoe (Lepidoptera: Crambidae), on maize (*Zea mays* L.) by the use of Napier grass (*Pennisetum purpureum* Schumach) as a trap crop and molasses grass (*Melinis minutiflora* P. Beauv.) as an intercrop [1,3]. In this system the adult females prefer to lay their eggs on Napier grass and are repelled by molasses grass leading to lower oviposition on maize. A similar push-pull approach was evaluated for *Eldana saccharina* Walker (Lepidoptera: Pyralidae) management in sugarcane where the determination was that adult female moths were not strongly attracted or repelled by the plants tested; these plants were not recommended for use in a push-pull management approach [4]. Thus, in the case of lepidopteran pests, the key to the success of this strategy lies in the oviposition preferences of the adult females. The trap crop should be preferred for oviposition over the main crop and the intercrop should be an oviposition deterrent which, upon contact, will stimulate movement elsewhere for oviposition [5]. However, larval mobility has important implications for pest management strategies that involve polyculture, such as trap cropping or a push-pull system. Ideally, larvae remain and feed on the trap crop and the main crop receives little or no damage. If the larvae, as well as the adults, prefer the trap crop to the main crop, then this could enhance the efficacy of the push-pull system by increasing tenure times and decreasing larval movement within the trap crop [6]. However, larvae are capable and often stimulated to move between plants [7,8]. So, even if adult females lay their eggs on the trap crop, subsequent movement of larvae into the adjacent main crop could minimize the effectiveness of a push-pull strategy. Although oviposition preference is crucial for the success of this pest management strategy, the behavior of larvae also has to be considered, especially if this is the stage when damage is inflicted, as is the case with many lepidopterans [9].

Pickleworms, the larvae of *Diaphania nitidalis* Cramer (Lepidoptera: Crambidae), are a pest of cucurbits found in Hawaii, North America (primarily in the Southeastern United States), Central and South America, and the West Indies [10,11,12,13,14]. Pickleworms feed on the fruits, buds, flowers, and stems of cucurbits [10,11,13]. Newly-hatched larvae feed on vegetative tissues, buds, and flowers, while older larvae tend to feed on fruits or tunnel the stems if the former are unavailable [12,13]. Pickleworms are primarily managed through weekly applications of insecticides and such practice is generally considered unsustainable. A potentially sustainable and environmentally sound management strategy for this pest could include push-pull cropping. We propose a potential push-pull system for pickleworm management on cantaloupe using squash (*Cucurbita pepo* L.) as a trap crop and watermelon (*Citrullus lanatus* [Thunb.] Matsum. and Nakai) as an intercrop. Squash was selected as a trap crop because it has been suggested that the use of squash as a trap crop (“pull”) might lower the risk of damage by pickleworm to other cucurbit crops [10,11,15,16]. Adult females have been shown to preferentially lay their eggs on squash plants [17,18] but field trials using squash as a trap crop have had variable success in reducing pickleworm damage to other cucurbit main crops [10,11,12,16]. It may be that even though there is a preference for oviposition on squash over cantaloupe that larval movement leads to reduced success of this strategy. In our study, watermelon was selected as an intercrop (“push”) primarily because it has typically had very low pickleworm damage despite being a cucurbit [18,19]. This could be because the adult moths lay a relatively low number of eggs on this plant. In addition, other factors, such as larval movement off the plant, could also contribute to the low level of damage seen.

In this study, we investigated the behavior of neonate pickleworm larvae, in relation to the potential push-pull cropping approach. Understanding the behavior of neonates is important to evaluate the potential of the proposed push-pull system. There may be various stimuli for neonate larval movement including crowding, reduced food resources or quality, presence of predators or parasitoids, microclimate preferences and feeding preferences [7]. If neonate larvae present on a squash plant (pull/trap crop) are stimulated to move and in their travel encounter either a cantaloupe or a watermelon plant. Their acceptance of either of these as a food plant will influence whether they remain on that plant or whether they continue to search for another host plant. The same would be true for a larva moving from a watermelon or a cantaloupe. Thus, understanding such larval preference could influence the effectiveness of a push-pull approach for management where the aim is to create a more complex habitat to influence pest behavior [4,9].

Furthermore, there may be an influence of early feeding on subsequent host plant choices should the larvae migrate away from its natal host plant. Lepidopteran larvae may acquire an induced preference after feeding on a particular host plant [20]. Experience has been shown to influence subsequent feeding activity. For example, after having eaten cabbage through its fourth instar, *Pieris brassicae* L. (Lepidoptera: Pieridae) larvae choose to starve to death rather than to feed on another host plant, nasturtium (*Tropaeolum majus* L.) [20]. The same phenomenon was found true for *Pieris rapae* L. (Lepidoptera: Pieridae) [21]. Thus, if an adult female pickleworm lays eggs on her preferred host plant and the larvae feed on that plant, it could become preferred over other plants and influence subsequent choices if the larvae left their natal host plant. Such a preference based on experience would be beneficial if the eggs were originally laid on the trap crop and the neonates then would choose squash over cantaloupe. However, this experience-induced preference could be damaging if it keeps any larvae within the main crop instead of traveling to the trap crop if they are stimulated to move.

There are other factors that will influence the success of neonate interplant movement and, hence, their interaction with the habitat. First, in spite of their small size, neonate larvae must be capable of moving relatively great distances. The mobility of neonate lepidopteran larvae has been previously described. For example, young larvae of the diamondback moth (*Plutella xylostella* L. (Lepidoptera: Plutellidae)) are capable of movement off their natal plants to adjacent ones [8]. Such behavior has also been described in the neonate larvae of the light brown apple moth (*Epiphyas postvittana* Walker (Lepidoptera: Tortricidae)) [22]. Foster and Howard concluded that these neonates are very mobile and their preferences have more influence than the adult female oviposition preference on the selection of the plants on which the larvae subsequently feed [22]. Although neonate pickleworms are small, around 1.5 mm in length, we observed in the lab that they are highly mobile; moving across lab counters and up walls in search of food after hatching from a cage in which no food was available to them. We observed that newly hatched neonate larvae could easily transverse the distance of 30 cm in under an hour, while walking on a flat piece of poster board. Another factor that will influence success of neonate mobility is their longevity without food. If larvae are without a food source they will eventually starve if they do not find a suitable food source and this is likely to be of critical importance for neonates where there are limited energy reserves. In the case of pickleworm, the longevity of starved neonates is unknown.

The first objective of this study was to gain a better understanding of the biology of neonate larvae; particularly the longevity of newly-hatched neonates without a food source to gain a better sense for how long a neonate could live while searching for a host plant or switching host plants instead of feeding. We conducted an experiment to examine the longevity of neonates when completely starved and after having limited access to food (24 h).

The second objective was to investigate if naive neonate larvae have a preference between host plants and if this preference can be modified through experience. To address the second objective a series of experiments were conducted to (1) see if naive neonate larvae had an orientation or settlement preference between squash, watermelon, cantaloupe, bean (non-host), and an artificial diet; and (2) to determine if experience feeding on one of the above food sources would influence the neonates’ subsequent orientation and settlement preference between the five food sources.

## 2. Materials and Methods

### 2.1. Insect Colony

Experimental larvae were obtained from a laboratory colony of pickleworm maintained at the University of Hawaii at Manoa, HI, USA. This colony was initiated in spring 2011 and sustained for two years. Larvae collected on the island of Oahu HI, USA from kabocha, zucchini, cucumber, and cantaloupe plants, were used to start and maintain the colony. The colony was keep at room temperature (22 °C) and exposed to natural day length.

Pickleworm larvae in the colony were reared on a pinto bean-based artificial diet (Bioserv Black Cutworm Diet, Newark, DE, USA). Once the larvae had finished feeding and were ready to pupate, indicated by a color change from greenish to pink, they were removed from the diet and placed into a separate container with paper towels, which they used to create a cocoon. After a few days, the pupae were collected and placed directly into the adult cage for emergence. The adult cage comprised of a fabric mesh flight cage (70 cm × 35.5 cm × 35.5 cm; BioQuip Products, Inc., Compton, CA, USA) in which the adults were provided with a 10% honey water solution. To provide an adequate level of humidity for oviposition, the cage was periodically sprayed with water and covered with white plastic trash bags. At any given time, there were up to several hundred adults in the cage of varying age and different generations, and they were allowed to mate freely. Cotton pads were hung from the top of the cage for the females to lay their eggs on, the cotton pads with eggs were then removed from the colony and placed in a glass jar. The newly-hatched neonates were obtained and used in the following experiments. The experiments were conducted over a period of two years, thus using neonates from different cohorts.

### 2.2. Neonate Longevity

To gather larvae for this experiment, unhatched eggs on cotton pads were in placed in plastic vials (3 cm diameter, 5.5 cm tall) with a plastic cap. After being placed in a plastic vial, the eggs within were given two hours to hatch, after which the newly hatched larvae were removed from the vial and used for the experiment. To examine neonate longevity, newly-hatched larvae were placed in one of two groups: “completely-starved” or “partially-starved”.

The neonates in the “completely-starved” group were placed in a plastic vial with no food. Larval survivorship was checked every 4 to 8 h until all larvae were dead. This was replicated fifty times. For each individual larva the total longevity was calculated as the time it was placed in the plastic vial to the last time-interval it was noted as being alive. In addition, the proportion of larvae that survived to each time interval was calculated.

The second group of “partially-starved” neonates was allowed to feed for 24 h and then placed in a plastic vial with no food, where their survivorship was checked every 4 to 8 h. To provide larvae with the opportunity to feed for 24 h each newly hatched larva was placed individually in a Petri dish (10 cm diameter × 1 cm deep) containing an l cm leaf disc of squash (*Cucurbita pepo* L. var. Cocozelle) atop moistened filter paper. This was replicated fifty-two times. For each larva the total longevity was calculated from the time it was placed in the Petri dish to feed to the last-time interval in the plastic vial where it was noted as being alive. Thus, for this group, the total longevity included the 24 h of feeding. The longevity post-feeding was calculated for the “partially-starved” group as the total longevity minus 24 h. In addition, the proportion of larvae that survived to each post-feeding time interval was calculated.

A two-sample *t*-test was used to calculate if there was a significant difference between the total longevity of the “completely-starved” and the “partially-starved” neonates. A second two-sample *t*-test was conducted to calculate if there was a different between the total longevity of the “completely-starved” group and the longevity post-feeding of the “partially-starved” group; thus comparing the longevity of both groups without access to food.

### 2.3. Naive Neonate Preference

Newly-hatched larvae were given a choice between different foods to investigate if they have naive orientation and settlement preferences. These preferences were determined by what food source the larva was on after 1 h (orientation preference) and after 24 h (settlement preference) of being presented with a choice of food sources. The larvae were obtained as described above. They were given a choice between potential foods: leaf discs of three host plant species in the plant family Cucurbitacae, a leaf disc from one non-host plant, and 1 cm disc of a pinto bean-based artificial diet (Bioserv Black Cutworm Diet, Newark, DE, USA). The two common host plants in Cucurbitacae were cantaloupe (*Cucumis melo* L.), and squash (*Cucurbita pepo* L.), and the third cucurbit, which is considered a rare host plant, was watermelon (*Citrullus lanatus* [Thunb.] Matsum. and Nakai) [11,18]. The non-host plant used was pole bean (*Phaseolus vulgaris* L.), in the plant family Fabaceae, because it has been reported that neonate larvae can feed on this plant species for 48 h without dying [18]. The plant varieties used were Atlantia Cantaloupe, Yellow Summer Crookneck Squash, Crimson Sweet Watermelon, and Emerite Pole Bean. These were selected because they are varieties grown in Hawai’i [23]. The artificial diet was incorporated because it had been used to maintain the pickleworm colony and had demonstrated itself as a sufficient diet for pickleworm development.

Each plant was greenhouse grown in an 8 cm square pot (9.5 cm depth) with Sunshine Mix 1 Professional Growing Mix (Pacific Agricultural Sales and Services, Inc., Kapolei, HI, USA) fertilized with 1.25 mL Osmocote Indoor and Outdoor Plant Food (Scotts-Sierra Horticultural Products Company, Marysville, OH, USA) and watered when needed, generally on a daily basis. The plants were between 27 to 31 days old when they were used in the experiments.

Leaf discs, 1 cm in diameter, were cut from leaves of each plant species. In addition, the artificial diet was prepared as a thin layer and cut in 1 cm discs. The leaf and diet discs were placed equal-distance apart from each other around the edge of a 10 cm diameter × 1 cm deep Petri dish in a random order to create a choice arena. To prevent the food discs from drying out, a moist piece of filter paper was on the bottom of each Petri dish.

To assess neonate preference one larva was placed in the center of each choice arena. The orientation preference of each larva was determined by recording the position of the larva after 1 h in the arena. The settlement preference of each larva was determined by recording the position of the larva after 24 h in the arena [9]. If a larva was not on a food source after 1 h it was recorded as a “no-choice” and was not used in determining the orientation preference. The orientation and settlement preferences for 50 naive neonate larvae were recorded.

### 2.4. Experienced Neonate Preference 

A second experiment was conducted to investigate if the orientation and settlement preference of the larvae is influenced by 24 h of experience feeding on a particular food source. This was performed as described above, except that once a larva hatched it was placed on one of the five food sources to feed for 24 h before it was placed in the choice arena and given a choice of all five food sources. For 24 h following hatching, each larva was placed on a 2.5 cm disc of one of the five food sources in a 5.5 cm × 3.5 cm × 3 cm plastic box and allowed to feed. The larva was then removed and placed in a choice arena with each of the five foods as described above. This was replicated at least 50 times for each of the five food sources.

For both the naive and experienced neonate preference experiments, a chi-squared test of goodness-of-fit test was used to find any unequal distributions between the larval orientation and settlement on the different food sources. If the chi-squared test indicated that there was not an equal distribution between the five food sources, then a subdivided chi-squares goodness-of-fit was done to determine if there was an equal distribution on the four plant species (the three cucurbits and bean). Finally, if there was an unequal distribution between the four plant species, then just the three cucurbit plant species were tested for an equal distribution.

## 3. Results

### 3.1. Neonate Longevity

Those larvae that were “partially-starved” lived longer than those that were “completely-starved” (*t*-value = 10.93, *p* < 0.0001, df = 85). The neonate larvae that were “completely-starved,” had an average total longevity of 42.48 h, with a range of 24 to 64 h. Those neonate larvae that were “partially-starved” i.e., they had 24 h of food access before being starved, had an average total longevity of 67.1 h, with a range of 40 to 112 h.

**Figure 1:**
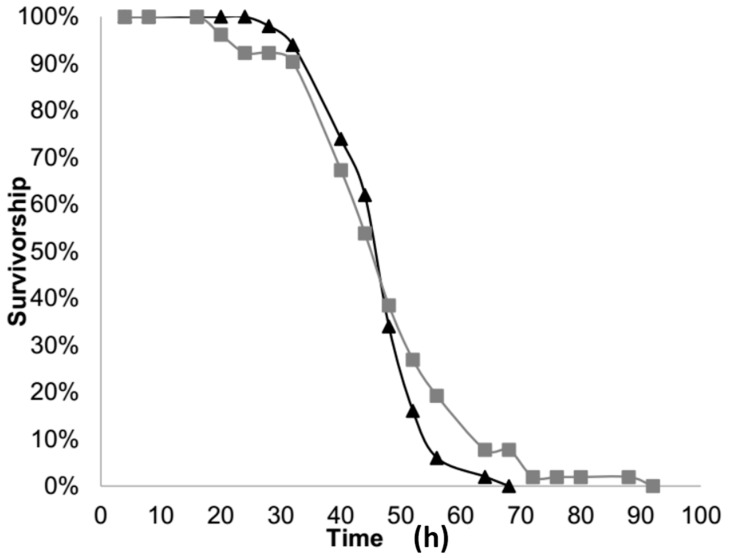
Percent of neonates surviving over time without food; triangles representing newly-hatched larvae (completely-starved) and squares representing larvae that had access to food for 24 h before starvation (partially-starved).

However, when comparing the longevity of larvae after both groups had no access to food (total longevity in the “completely-starved” group compared with the post-feeding longevity of the “partially-starved” group), there was no significant difference (*t*-value = 0.27 *p* = 0.791, df = 85). Once the larvae in the “partially-starved” group were removed from the food, they lived 43.1 h on average, with a range of 16 to 88 h and died as quickly as those that were completely starved (Figure 1).

### 3.2. Neonate Feeding Preference

#### 3.2.1. Naive Orientation

**Figure 2:**
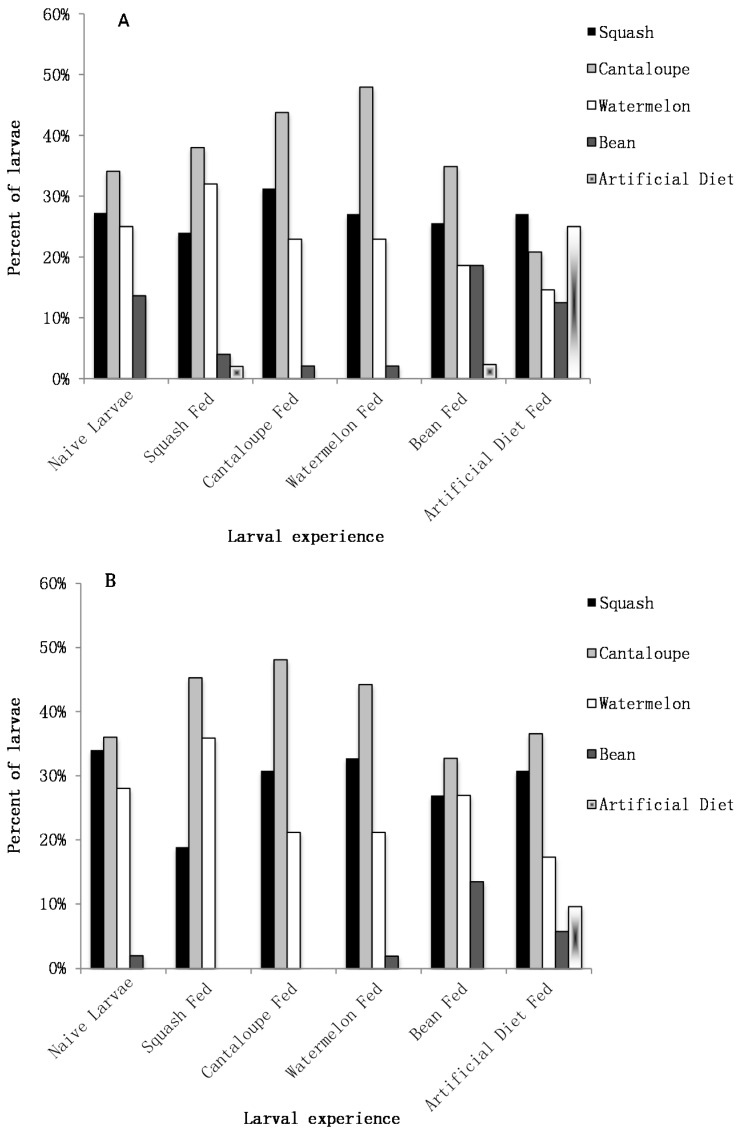
Percent of larvae (**A**) orienting to food sources after 1 h and (**B**) settling on food sources after 24 h following different feeding experiences.

An orientation preference of an individual larva was recorded based on the food source on which that larva was found on after 1 h following placement in the arena. In terms of orientation, the majority of naive neonate larvae (those with no feeding experience) were found on a food source within 1 h; only six larvae were recorded as a “no-choice.” The naive neonate larvae that were found on a food source after 1 h did not orient to all five food sources equally, X^2^ (df = 4, N = 44) = 15.77, *p* < 0.01. None of the larvae were found on the artificial diet; all were found on plant leaf discs. However, the number of larvae on each of the four plant species was not different, X^2^ (df = 3, N = 44) = 3.82, *p* > 0.05 (Figure 2A).

#### 3.2.2. Experienced Orientation

Experienced larvae were fed on one of the five food sources for 24 h prior to placement in the choice arena. Of the 53 larvae that had been fed squash before being put into the choice arena, 50 larvae were found on a food source after 1 h. The larvae did not orientate to all five food sources equally, X^2^ (df = 4, N = 50) = 26.6, *p* < 0.001, nor to all four plant species equally, X^2^ (df = 3, N = 49) = 13.45, *p* < 0.01. Only one larva orientated to the artificial diet and two larvae went to the bean. However, the remaining larvae did orientate to the three cucurbits equally, X^2^ (df = 2, N = 47) = 1.58, *p* > 0.05 (Figure 2A).

Of the 52 larvae that had been fed cantaloupe, 48 larvae were found on a food source after 1 h. The larvae did not orientate to all five food sources equally, X^2^ (df = 4, N = 48) = 34.08, *p* < 0.001, nor to all four plant species equally, X^2^ (df = 3, N = 48) = 17.67, *p* < 0.001. None of the larvae orientated to the artificial diet and only one larva went to the bean. The other larvae orientated to the three cucurbits equally, X^2^ (df = 2, N = 47) = 3.25, *p* > 0.05 (Figure 2A).

Of the 52 larvae that had prior feeding experience with watermelon, 48 were found on a food source after 1 h. The larvae did not orientate to all five food sources equally, X^2^ (df = 4, N = 48) = 37.42, *p* < 0.001, nor to all four plant species equally, X^2^ (df = 3, N = 48) = 20.33, *p* < 0.001. None of the larvae orientated to the artificial diet and only one larva went to the bean. However, the remaining larvae did orientate to all three cucurbit plant species equally, X^2^ (df = 2, N = 47) = 5.3, *p* > 0.05 (Figure 2A).

Of the 52 larvae that had been fed bean leaf before being placed in the choice area, 43 larvae were found on a food source after 1 h. The larvae did not orient to all five food sources equally, X^2^ (df = 4, N = 43) = 12.23, *p* < 0.025; only one larva was found on the artificial diet. However, the larvae did orientate to all four plant species (three cucurbits and bean) equally, X^2^ (df = 3, N = 42) = 3.14, *p* > 0.05 (Figure 2A).

During the orientation period, 48 of the 52 larvae that had been fed the artificial diet were found on a food source after 1 h. Larvae oriented equally to all the five food sources, X^2^ (df = 4, N = 48) = 3.88, *p* > 0.05 (Figure 2A).

#### 3.2.3. Naive Settlement

The food source that a larva was feeding on after 24 h of being in the choice arena with the choice of food discs was recorded as its settlement preference. All the larvae in this experiment were found on a food source after 24 h of being in the choice arena; therefore, no larvae were recorded as a “no-choice” for the settlement period.

The 50 naive larvae used in this experiment did not settle equally on all five food sources, X^2^ (df = 4, N = 50) = 31, *p* < 0.001; none settled on the artificial diet as all were on a leaf disc. The larvae did not settle equally on the four plant species, X^2^ (df = 3, N = 50) = 14.8, *p* < 0.01, only one larva settled on the bean. However, the naive larvae settled on all three cucurbits equally, X^2^ (df = 2, N = 49) = 0.53, *p* > 0.05 (Figure 2B). Eight larvae moved from one food source to another between the orientation and settlement period.

#### 3.2.4. Experienced Settlement

As described above, all of the larvae in this experiment were settled after a 24-h period. The larvae that had prior experience feeding on squash did not settle on all five food sources equally, X^2^ (df = 4, N = 53) = 48.33, *p* < 0.001, nor all four plant species, X^2^ (df = 3, N = 53) = 25.26, *p* < 0.001; none of the larvae chose to settle on the artificial diet or the bean leaf; all settled on one of the cucurbits. However, the larvae settled on the three cucurbits equally (X^2^ (df = 2, N = 53) = 5.72, *p* > 0.05) (Figure 2B). Five larvae switched food sources between the orientation and settlement recording.

Larvae that had prior experience feeding on cantaloupe did not settle on the five food sources equally, X^2^ (df = 4, N = 52) = 44.35, *p* < 0.001, nor the four plant species, X^2^ (df = 3, N = 52) = 25.08, *p* < 0.001, because no larvae chose to settle on the artificial diet or the bean leaf, instead all were settled on one of the cucurbits. There was equal settlement on the three cucurbits, X^2^ (df = 2, N = 52) = 5.82, *p* > 0.05 (Figure 2B). Two larvae moved between food sources from the orientation to the settlement period.

The larvae that had experience with watermelon did not settle on all five food sources equally, X^2^ (df = 4, N = 52) = 38.38, *p* < 0.001, nor all four plant species, X^2^ (df = 3, N = 52) = 20.31, *p* < 0.001. None of the larvae settled on the artificial diet and only one larva settled on the bean, the rest of the larvae settled on one of the cucurbits. The three cucurbits were settled upon equally, X^2^ (df = 2, N = 51) = 4.24, *p* > 0.05 (Figure 2B). Four larvae switched food sources between the orientation and settlement period.

The larvae that had prior experience feeding on bean did not settle on all five food sources equally, X^2^ (df = 4, N = 52) = 18.19, *p* < 0.01; none of the larvae settled on the artificial diet. The larvae did, however, settle equally on all four plant species, which included bean, X^2^ (df = 3, N = 52) = 4.15, *p* > 0.05 (Figure 2B. Two larvae changed food sources between the orientation and settlement period).

The larvae with experience feeding on the artificial diet did not settle on all five food sources equally, X^2^ (df = 4, N = 52) = 18.38, *p* < 0.01, though five larvae did chose to settle on the artificial diet. This was the only time that any larvae chose to settle on the artificial diet. Larvae did not settle equally on the four plant species, X^2^ (df = 3, N = 47) = 13.17, *p* < 0.01, with only three larvae that settled on the bean. The remaining larvae settled on the three cucurbits equally, X^2^ (df = 2, N = 44) = 3.61, *p* > 0.05 (Figure 2B). Thirteen larvae switched food sources between orientation and settlement, nine of which left the artificial diet after the orientation period to settle onto a cucurbit.

## 4. Discussion

The longevity experiment demonstrated that neonate larvae are capable of living, on average, 42.48 h, and up to 64 h, without food. When larvae had access to food for 24 h they lived up to 112 h total, with an average of 67.1 h total. However, feeding for 24 h before starvation did not provide larvae any greater benefit once they no longer had access to food. Overall, the longevity of completely-starved or partially-starved neonate larvae indicates that they have enough energy to live for a brief period while in search of a favorable food source. In a cropping environment, it is recommended that squash rows be planted 3–4 feet apart and plants within rows be planted 24–30 inches apart [23]. Given their 1 to 2-day longevity without feeding, neonate larvae could possess sufficient energy reserves that might allow them to move between rows of plants, if they had some sort of stimulation to do so. The question then follows: if they do move, would they be discriminating about their destination and would experience influence that decision?

Neonate pickleworm larvae are able to discriminate and make a feeding choice. They prefer plant foods to a suitable artificial diet as well as differentiate and choose host plants, cucurbits, over non-hosts, as evidenced in the movement of neonates over the course of the feeding preference experiment. Even within the first 24 h after hatching, larvae will consider their available options and make clear feeding choices based on the food sources presented to them.

Nevertheless, there does not appear to be an innate larval preference or a significant discrimination between the cucurbits even after having a prior feeding experience with a particular cucurbit species. As with the naive larvae, experienced larvae that had fed on a cucurbit for 24 h, did not orient or settle more frequently on one cucurbit over another. Overall these results indicate that there is no neonate larval preference between squash, cantaloupe, and watermelon, regardless of experience. Unless there is some trigger for movement, neonate larvae are not likely to move from their natal plant. In terms of this potential push-pull cropping system, this suggests that neonate larvae are not likely to move from the trap crop, if that is where they hatched, to the main crop due to a feeding preference alone.

A somewhat surprising result was that the influence of prior experience with a non-host food led to a change in larval behavior for both the bean-fed and artificial diet-fed neonates. Larvae that had the opportunity to feed upon bean for 24 h chose bean as frequently as the cucurbit species during both the orientation and settlement period. This pattern of orientation was the same as naive larvae; but the settlement choice was different suggesting that experience modified the behavior. It appears that experience with a non-host plant can actually influence the neonates’ preference and surprisingly make them more likely to accept a non-host plant (on which they may not be able to complete their development) in the presence of host plants.

In addition, larvae that had fed on a pinto bean-based artificial diet behaved differently than their naive counterparts, as well as those larvae that had fed upon a plant food source. The naive larvae did not orient nor settle on the pinto bean-based artificial diet. However, after having feeding experience on the artificial diet the larvae oriented to all five food sources equally. Furthermore, this was the only time larvae actually chose to settle on the artificial diet. These results indicate that the experience of a non-host food source will increase the larval acceptance of that food source even in the presence of host plants.

While interesting, this behavior of accepting of non-hosts is unlikely to influence the effectiveness of a push-pull cropping approach. Although, there has been speculation that in other polyculture systems the acceptance of non-host plants might reduce the efficiency of a polyculture approach [8]. It was found that the diamondback moth “may lay a large percentage of their eggs on non-host plants in polycultures” and this, coupled with larval movement into the main crop, could lower the effectiveness of companion planting, intercropping, and under-sowing on that pest [8]. However, the occurrence of this problem has more to do with the oviposition behavior of the females rather than the ability of the larvae to move from a non-host to a nearby host plant. Another study, which investigated the oviposition preference of pickleworm, suggested that female pickleworms did not prefer to lay their eggs on the non-host plant bean [17]. In fact, the adult females seemed to avoid oviposition on bean leaves, laying more eggs on their cage rather than on the bean leaves [17]. Whereas, when offered plants of cantaloupe, squash, and watermelon, adult female pickleworms laid eggs on all three but appear to favor squash [17]. Therefore, even though neonate pickleworm larvae did choose to feed on bean in some instances, it is unlikely that their mothers would choose to place them on this plant to begin with.

## 5. Conclusions

Overall, neonate pickleworms seem to have energy reserves that could potentially enable them to move away from their natal plant. It appears that the neonates do not have a feeding preference for one host plant over another within the proposed push-pull cropping system using watermelon (push), squash (pull), and cantaloupe (main). Thus, if stimulated to move from any one of these host plants, if the encounter is with another cucurbit, then they will likely settle and feed on the new host plant. They are unlikely to move from one of these host plants based on feeding preference. Thus, larval feeding preference is unlikely to undermine adult female oviposition preference. However, while feeding preference may not drive neonate movement, there may be other factors that could stimulate larval movement, such as population pressure, crowding, or resource limitation. A study of diamondback moth found that 16% of neonate larvae placed on a cauliflower plant moved to an adjacent cauliflower plant after eight days [8]. Additionally, other lepidopteran larvae have been known to go through a pre-feeding movement phase after hatching [7] but this has not been investigated in pickleworm. These other factors that could stimulate the movement of pickleworm neonates and other larval stages should be investigated. It is important to take the behavior of the larvae into consideration alongside the female oviposition preference when developing pest management strategies that involve polyculture cropping systems.
